# The perceived knowledge of the menstruation cycle and adjustment of swimming sets by swimming coaches based on menstrual-related issues

**DOI:** 10.17159/2078-516X/2022/v34i1a13851

**Published:** 2022-01-01

**Authors:** N Marais, H Morris-Eyton, N Janse van Rensburg

**Affiliations:** Department of Sport and Movement Studies, Faculty of Health Sciences, University of Johannesburg, South Africa

**Keywords:** adolescent swimmers, swimming coach, female swimmer, swimming training, prescription

## Abstract

**Background:**

Menstruation is the recurring discharge of the endometrial lining of the uterus as menstrual blood and tissue. The menstruation cycle affects most adolescent females and, although largely overlooked, affects women participating in sports.

**Objectives:**

The aim of this study was to determine whether coaches were aware of their swimmers’ menstrual cycles and whether coaches considered this information when adjusting training sets.

**Methods:**

Within the case study, a partial mixed-method, sequential dominant status approach was used. Data were collected in the form of questionnaires, focus group discussions, and one-on-one interviews. Coaches’ awareness of their female swimmers’ menstrual cycles was based more on observation than communication from the swimmer.

**Results:**

Coaches explained that training is adjusted based on their observations, but whether this is being done correctly during the menstrual cycle requires more research. Swimmers and coaches alike seem to have minimal knowledge of menstruation, its effects on training, and how to adapt to, or overcome, those effects during training or competition.

**Conclusion:**

In future, this knowledge could ensure the longevity of female swimmers in the sport. Understanding whether coaches and swimmers recognise the effect of the menstrual cycle within training and competition provides a more inclusive approach to ensure athlete longevity after puberty. This approach is grounded in creating an understanding between the swimmer and coach about the effect of menstruation during training and competition. It ensures an extended and more successful participation which may also assist in dealing with the ‘taboo’ surrounding menstruation and the female athlete.

Menstruation is the term used to describe the recurring discharge of the endometrial lining of the uterus as menstrual blood and tissue.^[1]^ Menstruation, however, is only one of several events that occur during what is known as the menstruation cycle. ^[2]^ The menstruation cycle is the time interval (counted in days) from the start of one menstruation to the start of the following menstruation, ^[3]^ and may vary in length depending on the individual. Regardless of the variability, normal cycles range from 21 to 34 days by the third year of menarche, ^[2]^ with the average length between cycles being 28 days. ^[1]^

Although the constant change in hormone levels during the menstrual cycle is not visible, the effects of these changes can be felt by those who menstruate. The period itself may be expected to bring about the most discomfort, due to the discharge of blood and the use of menstrual hygiene products such as tampons, menstrual cups, or sanitary pads. It is, however, during the days prior to menstruation that women report feeling the most discomfort due to pain, heaviness, fatigue, irritability, and lack of concentration. ^[4]^ Although the constant change in hormone levels combined with premenstrual syndrome (PMS) may become a part of everyday life for the general female population, it is important to examine whether these symptoms influence the female athlete during training and competition. Current national and international studies have attempted to understand and gain more insight regarding menstruation ^[5]^ and have also attempted to determine the perceptions about menstruation from males and females ^[5]^. However, research on the physical and emotional symptoms that women experience when menstruating and the effect that these symptoms have on sport performance is lacking.

The unique physiology of female athletes may require tailored training approaches that differ from those of their male counterparts. ^[6]^ Female physiology, such as menstruation and its relationship to training, have been discussed among female athletes and coaches in practice. ^[6]^ Since 2016, the world has begun to embrace the distinction between the sexes in sport, implying that men and women should be trained differently, based not only on hormone types and levels, but the respect of the emotional and physical effects that hormonal fluctuations can cause. ^[6]^ A study by Bruinvels et al. ^[7]^ found that more than half of elite female athletes in various sporting codes reported that their hormonal fluctuations during menstruation had a negative impact on their performance in training and competition. Yet Ihalainen ^[6]^ indicates that Olympic gold medals have been won by elite female athletes while menstruating during competition. Research by Bruinvels et al. ^[7]^ supports that of Martin et al. ^[8]^, having found that 77% of female athletes experience negative side effects, such as PMS symptoms, due to menstruation. These physical symptoms include back pains, cramps, headaches, and bloating. ^[8]^ This is further supported by Oosthuyse et al. ^[9]^ whose findings indicated that fluctuations in strength, metabolism, inflammation, body temperature, fluid retention and injury risk are associated with hormonal fluctuations during a female athlete’s menstrual cycle. However, it has been noted that little is known about how menstrual cycle-related side effects may or may not affect the female athlete’s performance, and how these effects differ between individuals. ^[8,9]^ The potential effects of hormonal fluctuations throughout the menstrual cycle on various types of training and adaptations thereof are as follows:

## The follicular phase

During the early follicular phase (Days 1 to 7 of the menstrual cycle), the female body is primed for high intensity exercise due to increased pain tolerance and higher perceived energy levels. ^[6]^ However, during the late follicular phase (Days 8 to 13 of the menstrual cycle), the rise in oestrogen hampers pre-exercise carbohydrate storage and therefore endurance female athletes experiencing this phase should increase carbohydrate intake the day before and during exercise to exercise at high intensities. ^[10]^ It has also been suggested that strength training may be more effective during the late follicular phase (Days 8 to 14 of the menstrual cycle or until ovulation). ^[10]^

## Ovulation

During ovulation (approximately Day 14 of the menstrual cycle), it is suggested that maximum strength could be achieved during menstruation rather than during ovulation ^[11]^. According to Hansen et al. ^[12]^, increased risk of injury in young active females may be due to a physiologically high concentration of oestrogen, which reduces fibrillar cross-linking and enhances joint laxity. No research has specifically noted increased swimming injuries during ovulation.

The menstrual cycle is part of a bigger health issue for female athletes. Low energy availability because of unbalanced energy intake and expenditure, which can be due to overtraining or eating too little, can lead to irregular periods, amenorrhea, or problems with bone health. ^[6]^ According to Mountjoy et al. ^[13]^ relative energy deficiency in sport (RED-S) affects, among other factors, menstrual functions in athletic girls, which also relates to the medical condition referred to as the ‘Female Athlete Triad’. The prevalence of menstrual disorders in sports, such as swimming, ranges from 16% to 82%. ^[14]^ Previous studies have found that female swimmers appeared to be more vulnerable to delayed puberty and menstrual irregularities because of inadequate body fat stores and exercise stress. ^[14]^ By understanding the emotional and physical effects of the menstrual cycle, it may be possible to determine at which stage training should be adjusted to optimise performance, based on the needs of the female athlete. The correct maintenance of training around the menstrual cycle could possibly assist in attaining peak performance and encourage female athletes to extend their participation in sport. Swimmers and coaches alike seem to have minimal knowledge of menstruation, its effects on training, and how to adapt to or overcome these effects during training or competition. Therefore, the aim of this study was to determine the female swimmer’s and swimming coaches understanding of the menstruation cycle and whether coaches considered this information when adjusting training sets.

## Methods

A partially mixed method, concurrent dominant status (qual + QUAN) design approach was used. This allowed a deeper understanding of menstruation from coaches and to give the participants a voice. Data were collected concurrently with the semi-structured interviews (swimmers and coaches) being done during the same time as the distribution and collection of the questionnaires. Questionnaires were completed by coaches and swimmers. They were either self-administered paper questionnaires or completed online.

### Theoretical framework

The study was underpinned by Brofenbrenner’s 1979 Ecological Systems Theory (EST) which was originally developed to focus on childhood development, examining how different environmental systems influence the development of an individual. This could be translated into the sporting environment and the athlete’s development within a particular sporting code. The EST explores the individual within a community, and the relationships that take place between them ^[15]^. It is through the community that the individual interacts. Although swimming is considered an individual sport, it takes a community to organise and develop a swimming programme. Coaches work closely with the athletes and parents, as well as the club, district, and provincial structures within the national federation (Swimming South Africa). It can be assumed that in the development of the athlete, each of these systems have an important role to play, with coaches being an influential component in the athlete-coach relationship.

The athlete-coach relationship is pivotal in this research, as sharing issues regarding the effects of menstruation on training and performance requires trust, respect, and confidentiality. ^[16]^ The primary responsibility of a coach is to assist their athletes with training and competition performance outcomes, as well as recognising the emotional and physiological state of the athlete. ^[16]^ Understanding that swimming coaching is embedded within a national federation system (Swimming South Africa), supporting female athletes will provide opportunities to facilitate talent and athlete development.

### Ethical clearance

Ethical approval for the study was obtained from the University of Johannesburg’s Faculty of Health Sciences Research and Ethics Committee (reference: REC-01-179-2018). Permission to conduct the study was obtained from the head coach of the swimming school. Participation in the questionnaires were anonymous. The identities of the participants who participated in the interviews were kept confidential. Coaches were not informed of which swimmers or coaches took part in the study nor were they made aware of any responses received by swimmers or other coaches. As per the Declaration of Helsinki the wellbeing of participants took precedence over the research outcomes. Participants volunteered and were allowed to leave the study without consequence. Consent and assent were received in writing once participants had been made aware of what the research entailed, allowing participants to freely agree to take part.

### Participants

The swimming coaches were sampled on a voluntary basis. The coaches had to be coaching in South Africa and coaching adolescent female swimmers who had already begun their menstrual cycles. The group of swimmers was purposefully sampled by meeting the following criteria: registered swimmers with swimming South Africa, had already begun their menstrual cycle and had to be between the ages of 13 and 18-years-old. The five swimmers that took part in the focus group discussion/interview were registered with Central Gauteng Aquatics (CGA) as competitive swimmers and were coached by the same coach.

### Recruitment method

An email requesting voluntary participants was sent to the head coach of the club. The coach was then requested to forward the information to the parents of the adolescent females of menstruation age. If swimmers were then willing to take part in the study, their parents were requested to contact the researcher directly. The researcher’s details were provided in the email. Once the researcher had received contact from the parents of willing swimmers, consent and assent forms were sent to the parent and/or guardian.

### Quantitative data

The coaches’ questionnaire was developed and adapted from Johnson.^[17]^ The swimmer’s questionnaire was based on similar questions in the coaches’ questionnaire and was restructured to contextualise the swimmer. The menstrual health section of the questionnaire was developed and adapted from Hendrix (2010). ^[18]^ Validity was ensured by asking the relevant questions and determining whether the questions reflected the perceptions and knowledge of menstruation.

### Swimmers focus group and interview

Swimmers participated in one focus group and one interview conducted by one researcher. No parents or coaches were present during the interview allowing the participants to feel relaxed and open to the discussion. Focus groups were initially planned with a group of five swimmers. Unfortunately, due to unforeseen circumstances only three swimmers were available on the day the focus group discussion was scheduled. The remaining two participants were interviewed separately.

### Coaches’ interviews

Semi-structured interviews were used to determine the coaches’ perspectives regarding the effects of menstruation on the female athletes they were coaching. There were six primary questions asked which were developed from the data collected from the coaches’ questionnaire. Two coaches were interviewed face-to-face by the researcher and four coaches were interviewed using the Zoom online platform, due to the limitations imposed by the COVID-19 pandemic.

### Data collection

The quantitative data were collected from May 2019 to May 2020. The coaches’ questionnaire consisted of 14 questions and there were responses from 31 coaches. The swimmer’s questionnaire consisted of 18 questions and 25 swimmers responded. Five swimmers took part in the semi-structured discussions, in which three questions were asked: (1) How do you usually feel when you have your period? (2) Do you think it affects your training? and (3) Would you discuss how you feel with your coach? The final phase of qualitative data collection was carried out by interviewing the swimming coaches. The semi-structured interviews were used to determine the coaches’ perspectives and consisted of six questions developed from the coaches’ questionnaire. Once all qualitative data had been collected, findings, including field notes and voice recordings, were transcribed to organise the data for analysis.

### Data analysis

After the questionnaires had been completed, separate Excel spreadsheets of findings from the coaches’ and swimmers’ questionnaires were created to organise the findings. The data were analysed using SPSS (version 26.0). Descriptive statistics and thematic coding of the qualitative data provided the analytical information from the data collected. Although quantitative and qualitative data were collected and analysed separately, the objectives of both were similar and therefore integrated into the results and discussion.

Data from the transcripts were divided into categories which were made up of different codes and sub-codes. The following categories were used for the swimmer focus groups and interviews: (1) Athletes’ perception of their period, (2) Athletes’ perception of the effects of menstruation on training, and (3) Athlete and coach relationship. Categories used for the individual coaches’ interviews included: (1) Coaches’ awareness of swimmer’s menstruation, (2) Swimmer-coach relationship (indicating that swimmers initiated the discussion around menstruation or menstrual-related issues with the coach), (3) Accommodating the needs of the swimmer in training, and (4) Coach-swimmer relationship (this indicates that the coach initiated conversations around menstruation or menstrual-related issues with the swimmer). Categories were determined by the overall themes of the swimmers, menstruation, coaches, and their relationships. Coding was done by one researcher and an independent coder who had no previous knowledge of the study. All themes, categories and codes were agreed to by the research team.

## Results

### Demographics

Twenty-five female swimmers participated in both the qualitative and quantitative parts of this study. Of those swimmers three were 13-years-old, six were 14-years-old, four were 15-years-old, one was 16-years-old, nine were 17-years-old and two were 18-years-old, respectively. The swimmers had a mean age of 15.5 years, with a standard deviation of 1.7 years. The majority (52%) of the female swimmers were South African National Junior (SANJ) swimmers, 12% were Level 3 swimmers, 20% Level 2 swimmers, and 12% were Open Water swimmers, with one swimmer not providing her swimming level. A total of 31 coaches completed the coaches’ questionnaire, of whom six coaches additionally took part in the semi-structured one-on-one interviews. Of the 31 coaches, 18 were self-identified females and 13 were self-identified males. In addition to the questionnaire, the six coaches who took part in the interviews comprised three males and three females.

### Awareness

Three questions in the swimmer’s questionnaire highlighted the coaches’ awareness as follows: (1) Is your coach aware of when you menstruate? (2) Does your coach ask you when you started menstruating?, and (3) Does your coach speak to you about menstrual-related issues/topics? Most of the swimmers (20 of the 25) indicated that their coach was not aware of when they were currently menstruating. While 19 of the 25 swimmers indicated that their coach does not ask when they started menstruating, and 18 stated that they do not discuss menstrual-related issues with their coaches. However, the majority (20 out of 31) of the coaches indicated that they make a point of asking if their swimmer has reached menarche. When asked if they are aware when their swimmers are menstruating, 87% of the coaches stated that they are aware of when their swimmers are currently menstruating.

### Consideration and training set adjustments

Of the five swimmers who said that their coach was aware of their menstruation, three admitted that their coach does accommodate them during training when they are menstruating by being more patient and sympathetic or by making the training session easier. Two of the girls said that their coach did not accommodate them. All but one of the coaches said that swimmers request to withdraw from swimming training or competition because of menstrual-related issues; however, only 22 coaches consequently allowed their swimmers to withdraw. In addition, most of the coaches (30 out of 31) believed that the menstrual cycle has the potential to influence sports performance, but only 22 stated that they changed their expectations of the swimmer if they became aware that she is menstruating or suffering from menstrual-related issues. When asked, only 18 of the coaches were willing to adjust training sets if their swimmer is menstruating or suffering from menstrual-related issues. During the interviews, the majority (five out of the six) of the coaches said that they only adjust training sets if the swimmer is experiencing severe signs or symptoms of premenstrual syndrome or menstruation, such as ‘heavy periods’ and ‘high pain levels’. Three of the coaches further mentioned that they will stop the training session if the swimmer is not coping. Two of the coaches, both female, mentioned that they do not adjust sets and avoid drawing attention to the swimmers if they are menstruating.

## Discussion

Perceptions, awareness, and knowledge around menstruation and the menstrual cycle varied between coaches and swimmers. Swimmers do not believe that their coaches are aware of their menstruation, while coaches believe that they are. This contradiction highlights the fact that the coaches’ awareness of their swimmer’s menstruation was based more on assumption than determining the facts. The lack of communication and knowledge regarding the female athlete’s state for training and competition compromises the strength of the coach-athlete relationship.

When exploring awareness of menstruation and menstrual-related issues, the swimmers’ and coaches’ results varied, reflecting different perceptions among the two groups. When coaches were asked about their awareness of their swimmers' menstruating, only fourteen indicated that the athlete made them aware of this, while the majority of swimmers indicated that their coaches were not aware of their menstruation status. In addition, nineteen of the swimmers stated that their coach does not ask them about menstruation or menstrual-related issues. The coaches' answers in the questionnaire highlighted an opposing opinion of the relationship, where twenty of the coaches indicated that they had an open discussion with their swimmers in which they asked them whether they had started menstruating. However, when interviewed, all the coaches said that they do not ask their swimmers about menstruation or menstrual-related issues only speaking about the topic if the swimmer approaches them about it. Research conducted by Johnson ^[17]^ found that 0.7% to 1.5% of coaches of various sporting codes asked their athletes about menstruation. The data obtained from the one-on-one interviews concur with this result. This necessitates a need to be a clear understanding between swimmers and coaches that menstruation does not imply weakness or that the periodised training plan needs to change. It is, however, an opportunity for the coach to track whether they are meeting the training needs of their female athletes to optimise training for the individual.

In general, the research is inconclusive and there are mixed reviews regarding the menstrual phases and their effects on performance. ^[8,9]^ Most females experience signs and symptoms of PMS, which can hamper an athlete’s performance. The PMS symptoms that the athlete experiences can make them hesitant to train or not want to train. ^[19]^ This should not be seen by coaches as a weakness in the athlete but rather as an opportunity for the coach to adjust training sets within the periodisation plan, such as working the athlete harder when they are not experiencing PMS symptoms and using the days when the athlete experiences their PMS symptoms as an opportunity to work on technique or recovery. Not only will the athlete be more willing to train, but the approach will be more inclusive, giving the athlete a psychological edge (the mental advantage of not being discouraged by their menstruation or PMS, e.g. the athlete will be willing to train with or without PMS symptoms) and enabling their training sessions to work for them rather than against them.

Speaking openly about the menstrual cycle should not be viewed as taboo or embarrassing or used as an excuse to avoid training. Rather it should be used to increase knowledge, empowering both the coach and the athlete. Open communication allows coaches and athletes to avoid early signs of RED-S from not tracking the menstrual cycle. ^[19]^ It will also assist coaches to monitor and track their female athletes, which, in turn, may encourage lifelong participation in sport and help an athlete progress in their sport as needed. ^[19]^ Communication between the coach and swimmer may assist in minimising the assumptions made by coaches about PMS symptoms experienced by athletes. Without assumptions, the coach can cater more accurately to the athlete’s needs, making the coaching process more individualised, considering all aspects of training, not just the programme and the competitions.

Consideration was given regarding whether coaches were aware of information concerning the swimmer’s menstrual cycles before exploring whether adaptations or adjustments were made by coaches to training sets based on the swimmer’s menstrual signs and symptoms. Although coaches claimed to be aware of a swimmer’s menstruation patterns, many swimmers indicated that coaches were not aware of this information. Further investigation through the interviews revealed that coaches do not ask and are not told by the swimmers about their menstrual cycles. Rather, awareness of their swimmer’s menstruation cycle is based on assumptions, such as the age of the swimmer or complaints of headaches, cramping and fatigue. Although these are all signs and symptoms of PMS, many other factors, such as illness, intense exercise, and dehydration can induce the same signs and symptoms. With the age of menarche continuously shifting and based on many factors, coaches can no longer use age as a defining factor that a female has in fact reached puberty.

According to the swimmers, if the coach was aware of their menstruation (five girls reported that their coaches were aware), only three of the five coaches accommodated the swimmer. It was, however, highlighted that accommodating the swimmer was not necessarily done by adjusting training sets, but rather by the coach being supportive and understanding of the swimmer’s circumstances. However, the coaches who answered the questionnaire gave a different interpretation. Most alluded to accommodating the swimmer and adjusting sets by making the sets shorter or less intense, or by letting the swimmer leave the pool if they were not coping in the training session. This contradicts the findings of Johnson ^[17]^ in which none of the coaches responded that they shortened sets or decreased their intensity for their female athletes experiencing menstrual-related issues. Only 0.8% of the coaches in Johnson’s ^[17]^ study worked out a plan to enable the athlete to cope during menstruation, and 4.6% allowed the athlete to decide whether they could train or not; however, approximately 40% of the coaches were empathetic. The difference in results could be due to the coaches in Johnson’s study coaching athletes at different school levels, whereas in this study, only registered competitive club swimmers were considered.

If coaches are more aware of their swimmers' menstrual cycles, they can periodise training and adapt their training sets in a way that would be beneficial to the performance of the swimmer. Stopping a set, decreasing the volume, or decreasing the intensity may not necessarily be the correct measure to put in place when a female swimmer is struggling with premenstrual or menstrual signs and symptoms. Although no significant effects of the menstrual cycle on performance have been recorded, some studies have shown that strength training, endurance sets or anaerobic sets at various phases within the menstrual cycle are beneficial to female athletes. ^[9,10]^ Muscle strength and muscle diameter have been shown to be increased during follicular phase-based strength training, more so than in luteal phase-based strength training, suggesting that strength training should be done during the follicular phase of the menstrual cycle ^[10]^. However, Oosthuyse et al. ^[9]^ found that endurance performance increased during menstruation and therefore athletes should be trained in the mid-luteal phase of the menstrual cycle. Whereas Pestana et al. ^[20]^ found no difference in anaerobic performance between the luteal and follicular phase; however, they noted that maximum heart rate was significantly lower in anaerobic performance during the mid-follicular phase. With that in mind, coaches would have an advantage if they planned their training sets according to their female athletes’ menstrual cycle.

### Practical applications

Communication between the swimmer and the coach is essential for the correct periodisation and training to maximise performance. Guidelines to assist with non-invasive and open communication include:

1. A blank monthly calendar in an online format (such as Google Docs) that is accessible to the coach and swimmer. The parent can be included, especially with swimmers who are minors.
2. Educating the swimmers on how to track their menstrual cycles. For example, marking the day their period starts and the day their period ends on a calendar.
3. Educating the coaches about the phases of the menstrual cycle according to what their swimmers have marked on the calendar.
4. Educating the coaches about the type of training that is most effective during each phase.
5. Explaining to the coach that they need to look for red flags, such as the absence of swimmer’s period over an extended time, or if the swimmer’s period is shorter each month.
6. The swimmer could supply other information, such as signs and symptoms, using journals or notes on calendar days, if they are willing.

The guidelines proposed above allow coaches to be aware of the swimmer’s menstrual cycle without the swimmer having to verbalise this information to the coach. To protect the swimmer’s privacy, consent for this would be given by the parent and assent by the swimmer. The document would also need to be password-protected to ensure that no one other than the three parties have access to the document.

### Limitations and recommendations

Female swimmers and the effects of menstruation cannot be generalised as each individual’s experience of the menstrual cycle may be different. This is an indication that each athlete should be treated and trained using an individualised tailored approach, especially in individual sports such as swimming and running. Coaches and athletes need to speak openly about menstruation, its effects and how to deal with it. By tracking the athlete’s menstrual cycle, the coach and athlete become more aware of the female physiology, ensuring that overtraining does not take place and giving the coach and the athlete an opportunity to work with the athlete’s menstrual cycle, rather than against it. Future research should include larger sample sizes from more swimming clubs across South Africa. Contraception use was not factored into this study, and it may have an influence on hormones and the swimmer’s experience of her menstrual cycle. For future research, it is recommended that contraceptive use versus no contraceptive is considered. The swimmer’s sporting career and how long they had been swimming were also not considered and could be beneficial for future studies to determine whether the duration of an athlete's career may affect their experience and perception of their menstrual cycle.

## Conclusion

Coaches and swimmers alike seem to have minimal knowledge regarding menstruation, its effects on training, and how to adapt to and/or overcome those effects during training or competition. Although the majority of the coaches adjust training sets if their female swimmers are experiencing menstrual related issues, how these adjustments are made and their effects on performance require more research. Having an enhanced understanding around the menstrual cycle and its effects on the female athlete could increase the longevity of swimmers within the sport. It would also allow for better swimmer management related to training and competition. Understanding whether coaches and swimmers recognise the effect of the menstrual cycle within training and competition provides a more inclusive approach to ensure athlete longevity after puberty. This approach is grounded in creating an understanding and developing a trusting relationship between the swimmer and coach regarding the effects of menstruation during training and competition. It may also ensure an extended and more successful participation in swimming, as well as assist in dealing with the ‘taboo’ regarding menstruation and the female athlete.
